# Supplementary material for: Dual blockade of CD47 and HER2 eliminates radioresistant breast cancer cells
Source: Nat Commun. 2020 Sep 14;11:4591. doi: 10.1038/s41467-020-18245-7 (PMC7490264; doi:10.1038/s41467-020-18245-7)
Supplement: Supplementary file 1 — Supplementary Information [file 41467_2020_18245_MOESM1_ESM.pdf]

Supplementary Information

Dual blockade of CD47 and HER2 eliminates radioresistant breast cancer cells

Candas-Green et al.

This file contains:

Supplementary Figures 1-10

Supplementary Table 1

Fig. S1

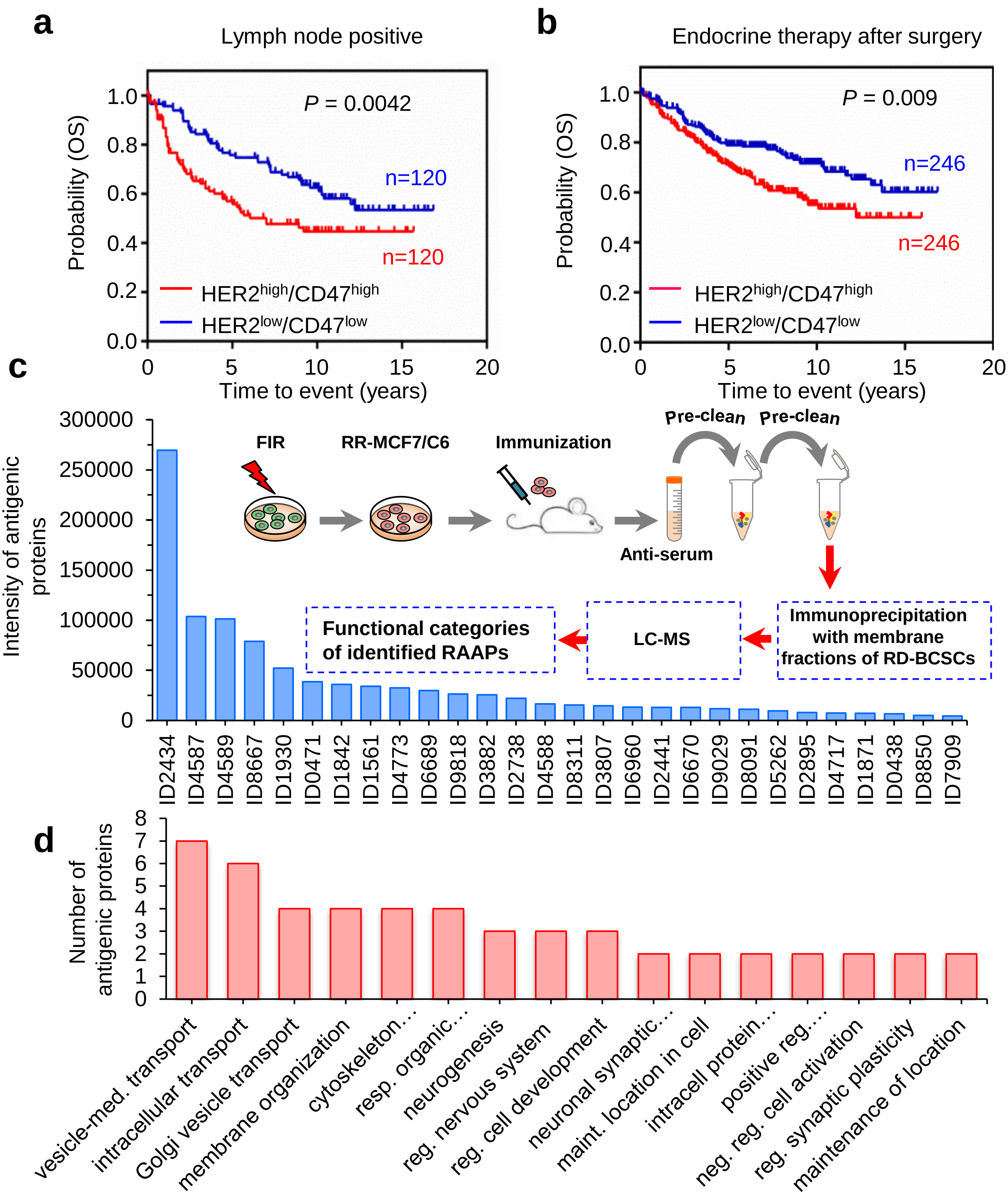

Supplementary Figure 1. (a) Co-expression of CD47 and HER2 in overall survival (OS) of BC patients with lymph nodes metastasis and (b) BC patients received endocrine therapy after surgery, from Breast Cancer Meta-base: 10 cohorts 22k genes database generated by SurvExpress (http://bioinformatica.mty.itesm.mx:8080/Biomatec/SurvivaX.jsp) from the HER2 probe 210930_s_at combined CD47 probe 211075_s_at. Statistical significance was analyzed by log-rank test. (c) Insert: scheme for generation of antiserum of radioresistant BC cells. A cluster of radiation associate antigenic proteins (RAAPs) including CD47 and HER2 were identified from the anti-serum of mice immunized with MCF7/C6 cells derived from radioresistant MCF7 cells. The RAAPs were detected through pre-cleaning via incubation with normal breast epithelial MCF10A and then breast cancer MCF7, and immunoprecipitated with membrane proteins of RD-BCSCs. The immunoprecipitated membrane proteins were analyzed with LC-MS and grouped with richness in expression. (d) Functional categories of identified RAAPs.

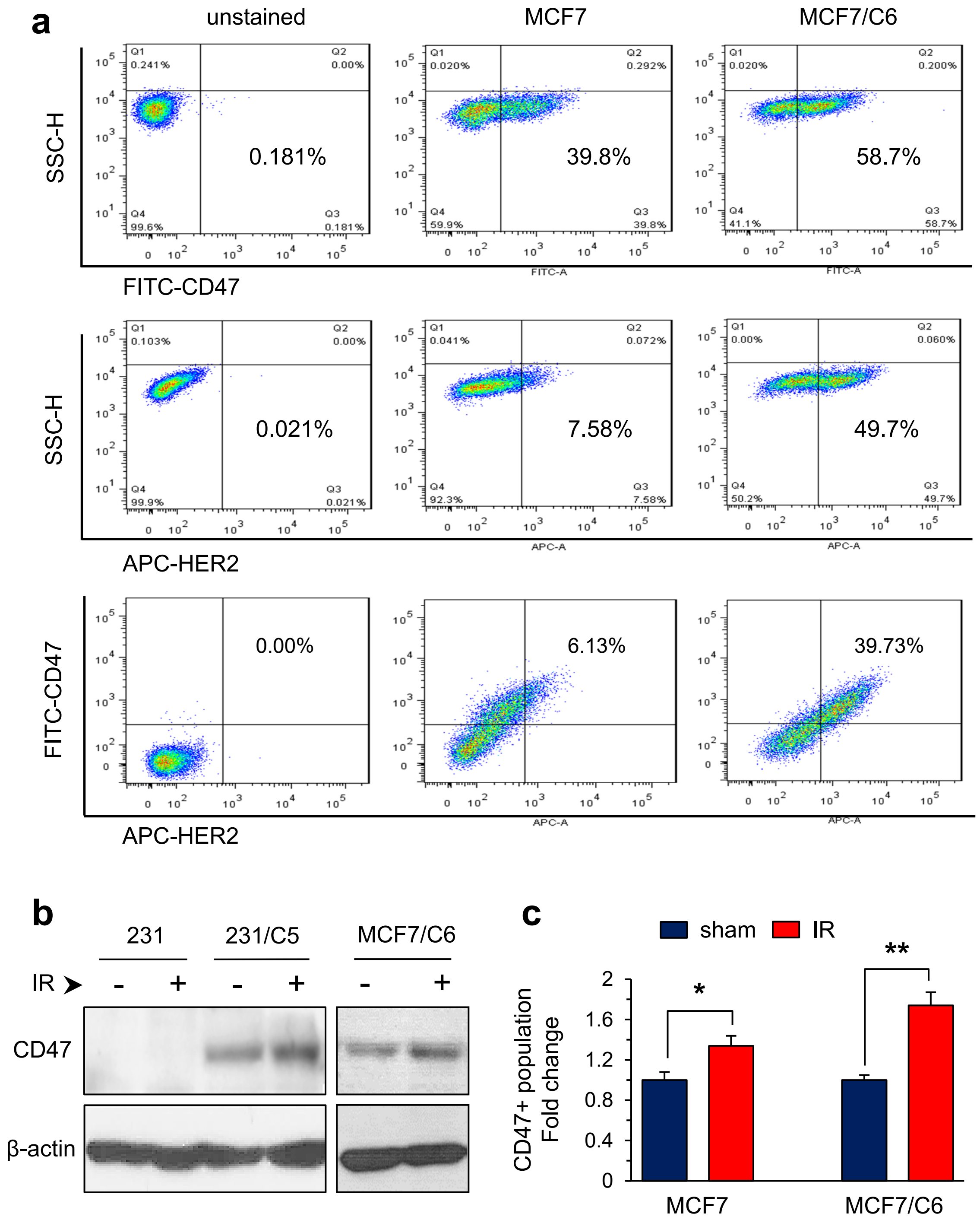

Supplementary Figure 2. Co-expression of HER2 and CD47 is enhanced in radioresistant BC cells.
(a) Flow cytometry analysis of $CD47^+$, $HER2^+$ or $CD47^+/HER2^+$ populations in wild type MCF7 and radioresistant MCF7/C6 cells. (b) Immunoblotting of CD47 in sham and IR-treated MDA-MB-231(231) and radioresistant MDA-MB-231/C5 and MCF7/C6 cells. (c) Flow cytometry analysis of $CD47^+$ population in MCF7 and MCF7/C6 cells with or without 5 Gy IR (n = 3; *$P < 0.05$, **$P < 0.01$).

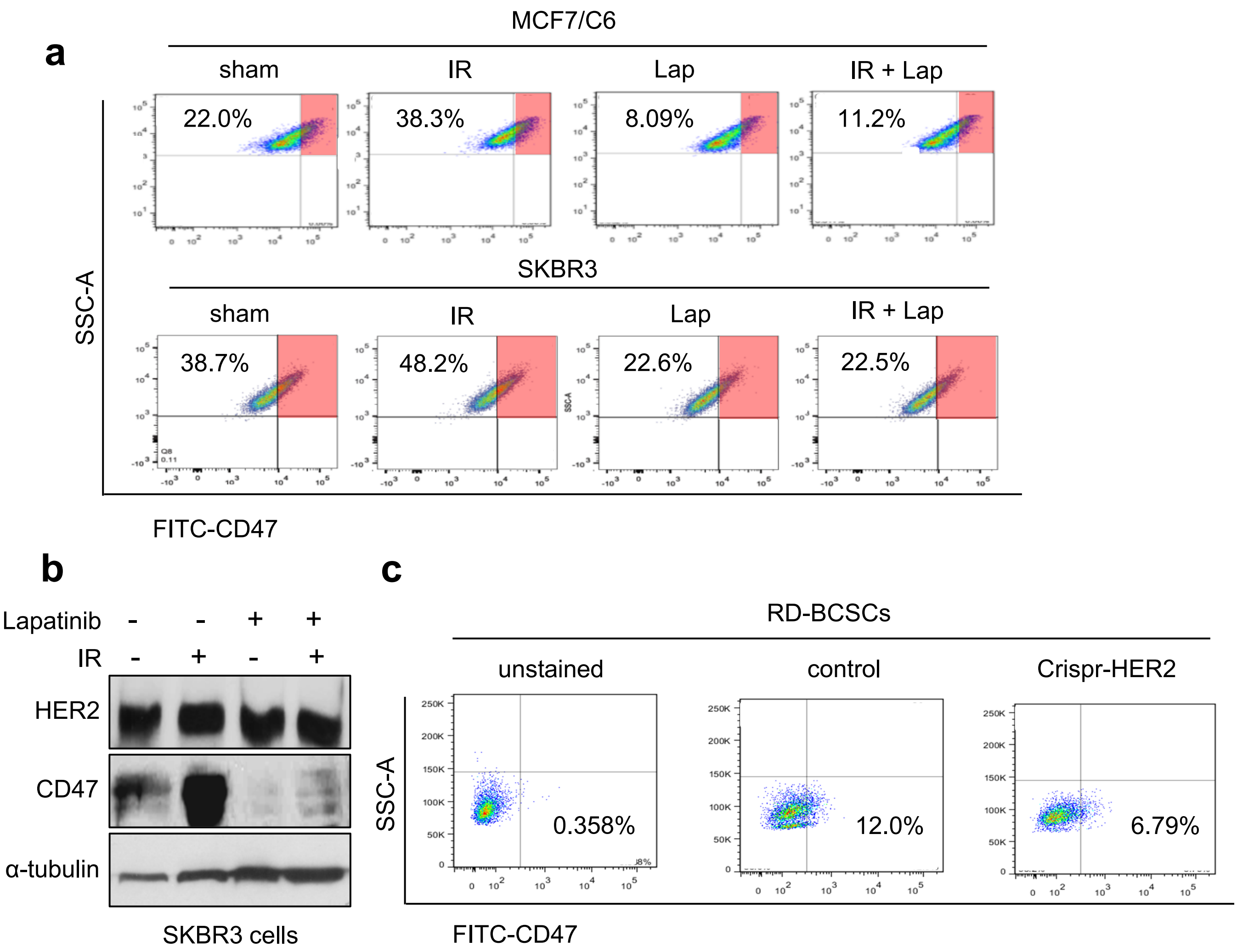

Supplementary Figure 3. Antibody blockage of HER2 or CRISPR/Cas9-KO HER2 reduces CD47 expression and CD47$^+$ cell population.

(a) Gating strategy for flow cytometry analysis of CD47$^+$ cells in MCF7/C6 or SKBR3 cells treated with Lapatinib, IR (5 Gy) or Lapatinib (10 μM Lapatinib for 72 h) followed by IR (n=3). (b) Immunoblotting of HER2 and CD47 in SKBR3 cells treated with Lapatinib, IR or Lapatinib followed by IR. (c) CD47$^+$ cells in HER2$^+$ RR-BCSCs and the counterparts of CRISPR-KO HER2.

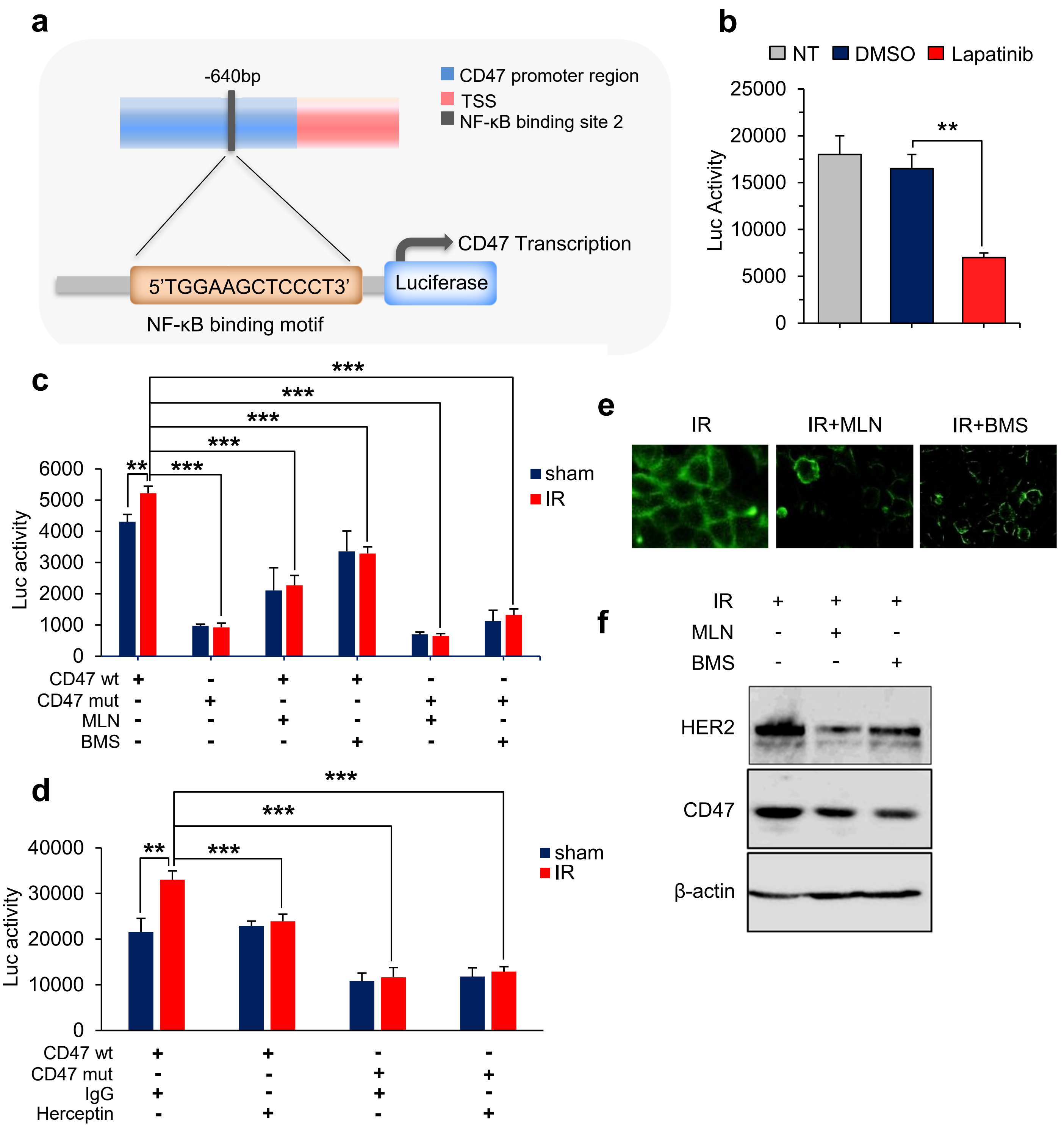

Supplementary Figure 4. NF-κB controls CD47 promoter transactivation. (a) Schematic of human CD47 promoter region near transcriptional starting site (TSS) with a putative NF-κB binding sequence -640bp (5'TGGAAGCTCCCT3', used for Luciferase reporter construction). (b) Human CD47 promoter region (-640bp) cloned into pGL2-basic plasmid and the CD47-luciferase activity was measured in MCF7/C6 cells with or without Lapatinib treatment. NT, no treatment; RLU (relative luciferase units; 10 μM Lapatinib for 72 h; DMSO as solvent control; n=3, **P < 0. 01). (c) CD47 promoter activity with wild type or mutant NF-κB binding motif measured in control and irradiated MCF7/C6 cells in the presence or absence of NF-κB inhibitor MLN120B (MLN, 20 μM) or BMS-34554 (BMS, 20 μM, 15 h; n =3, ***P* < 0. 01, ****P* < 0. 001). (d) CD47 promoter activity with wild type or mutant NF-κB binding motif measured in control and irradiated MCF7/C6 cells in the presence or absence of Herceptin (10 μg/ml, 3 days; n =3, ***P* < 0. 01, ****P* < 0. 001). (e) Representative images of immunofluorescence of CD47 in irradiated MCF7/C6 cells pretreated with MLN (20 μM) or BMS (20 μM), CD47 was visualized by confocal microscope 16 h after IR. (f) Immunoblotting of CD47 and HER2 in IR-treated MCF7/C6 cells with or without MLN (20 μM) or BMS (20 μM).

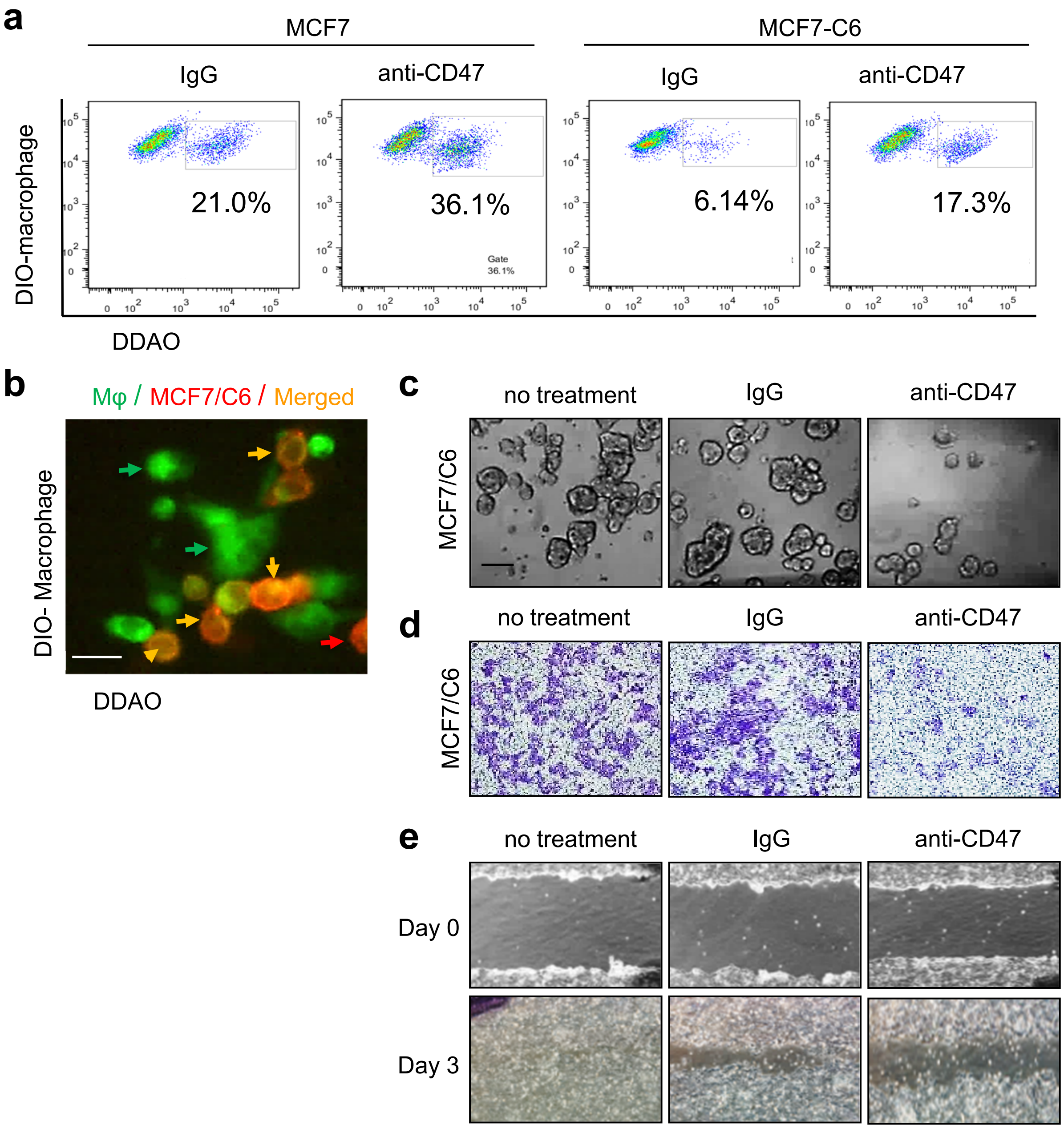

Supplementary Figure 5. Antibody blocking CD47 not only enhances macrophage phagocytosis but also inhibits in vitro cell aggressive growth.

(a) Flow cytometry analysis of macrophage phagocytosis on MCF7 and MCF7/C6 cells with or without antibody blockage of CD47. (b) Representative immunofluorescence staining of macrophage-mediated phagocytosis; orange color represents MCF7/C6 cells (red, DDAO) phagocytosed by a mature macrophage (derived from green THP1, DIO; scale bar, 25 μm). The anti-CD47 mediated inhibition on in vitro aggressive growth of MCF7/C6 was detected by mammosphere formation (c), transwell invasion (d) and gap filling assay (e) with no treatment and IgG included as controls; scale bar = 100 μm.

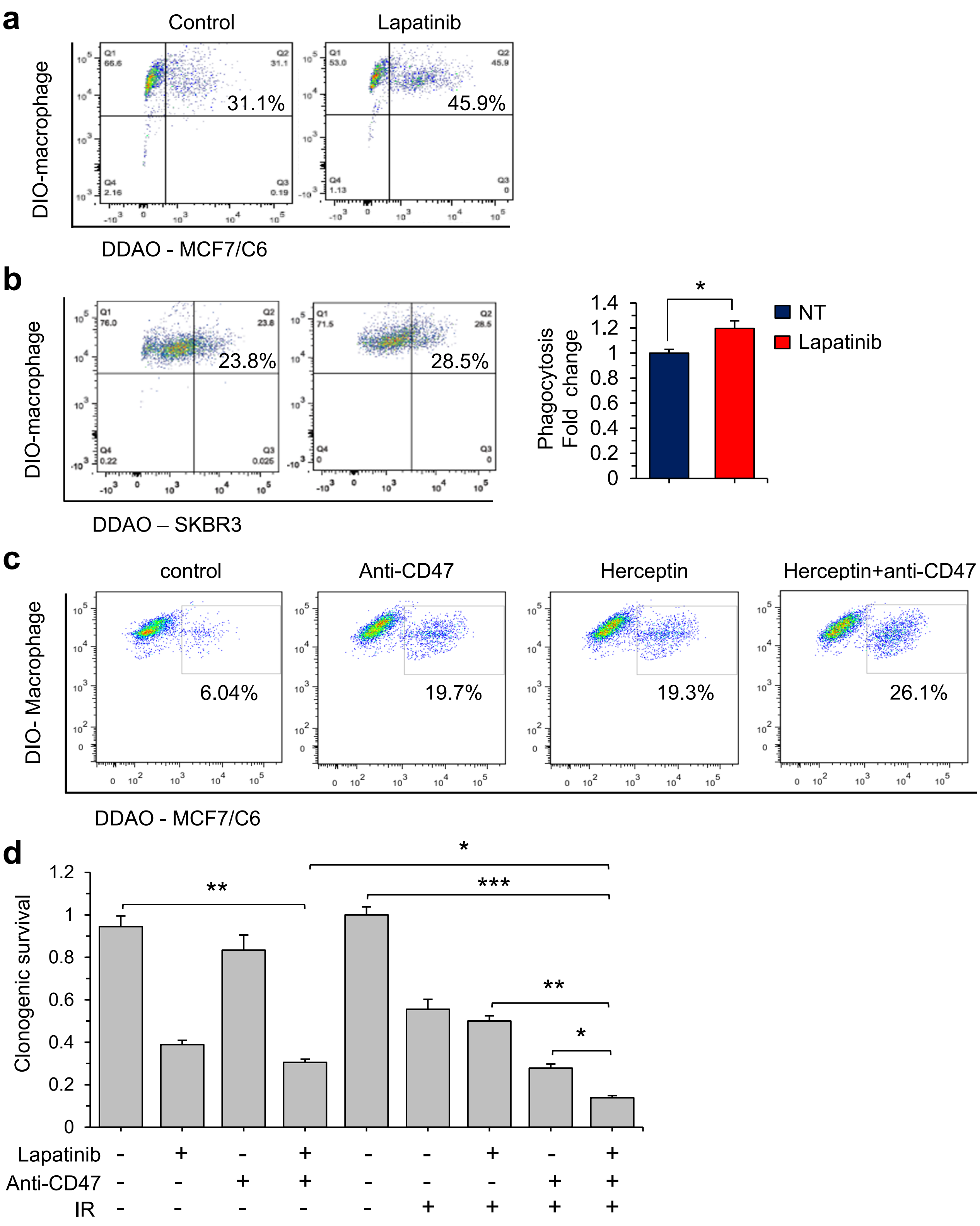

Supplementary Figure 6. Antibody blocking HER2 enhances macrophage phagocytosis and radiosensitizes cells.

(a) Gating strategy for macrophage phagocytosis on MCF7/C6 cells with or without HER2 blockage by Lapatinib. (b) Enhanced macrophage phagocytosis on HER2-expressing SKBR3 cells treated with Lapatinib measured by flow cytometry and quantified in right (n = 3; **P* < 0.05). (c) Flow cytometry analysis of enhanced macrophage phagocytosis on MCF7/C6 cells treated with combination of Herceptin and anti-CD47. (d) Clonogenic survival of HER2-expressing SKBR3 cells treated with different combinations of anti-CD47, Lapatinib, and IR (IgG and anti-CD47 antibody were applied with 10 μg/ml for overnight, Lapatinib was applied with 10 μM for 72 h prior to survival assay; IR = 5 Gy (n = 3, **P* < 0.05, ***P* < 0.01, ****P* < 0.001).

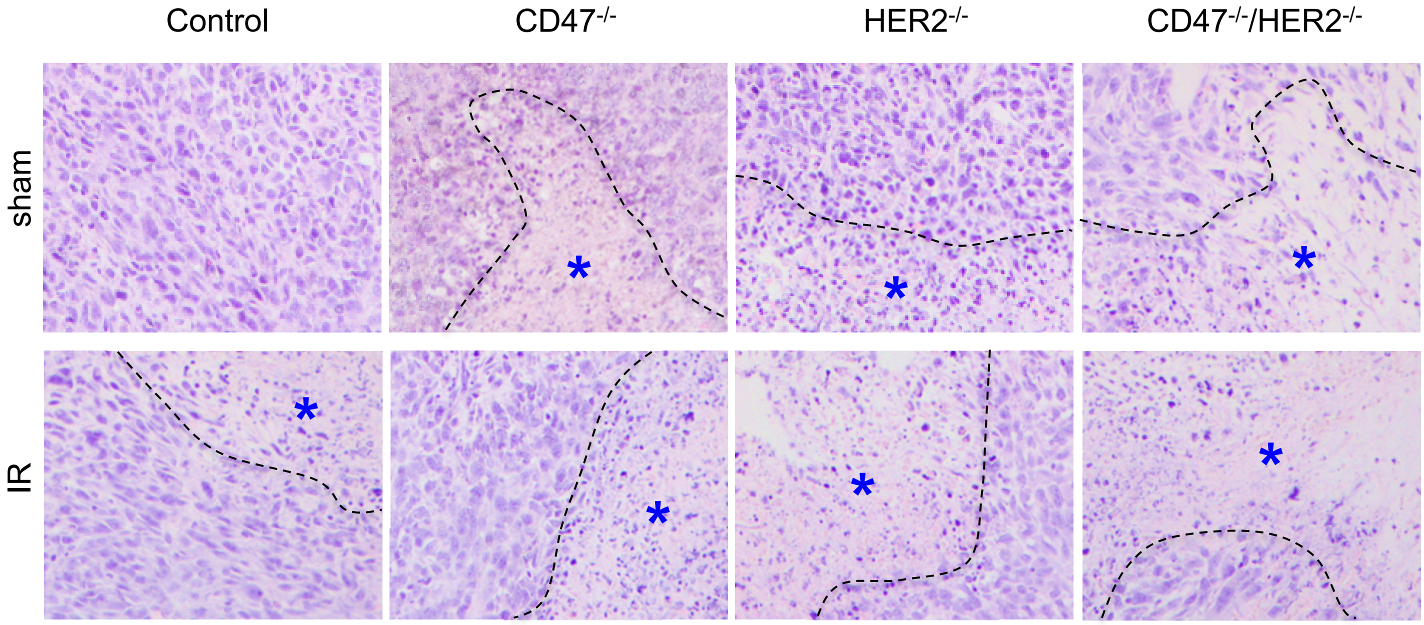

Supplementary Figure 7. Histological features of CD47$^{-/-}$, HER2$^{-/-}$, CD47$^{-/-}$/HER2$^{-/-}$ tumors. Pleomorphic tumor cells with obvious mitoses were detected in control tumors, whereas less proliferative feature and increased necrosis and apoptosis were detected in the CD47$^{-/-}$, HER2$^{-/-}$ tumors and mostly enhanced in the CD47$^{-/-}$/HER2$^{-/-}$ tumors. The necrosis areas were circled by dash line and marked with an asterisk.

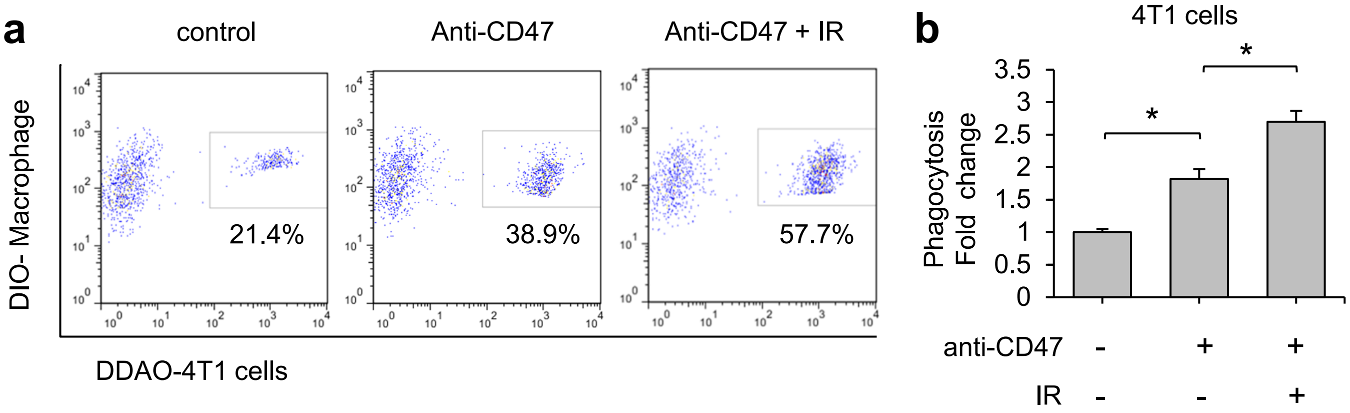

Supplementary Figure 8. RT combined with antibody blocking CD47 enhances macrophage phagocytosis.
(a) Flow cytometry analysis of macrophage-mediated phagocytosis on mouse breast cancer 4T1 cells treated with anti-CD47 antibody with or without 5 Gy IR. (b) Quantitation of A, n = 3 *P < 0. 05.

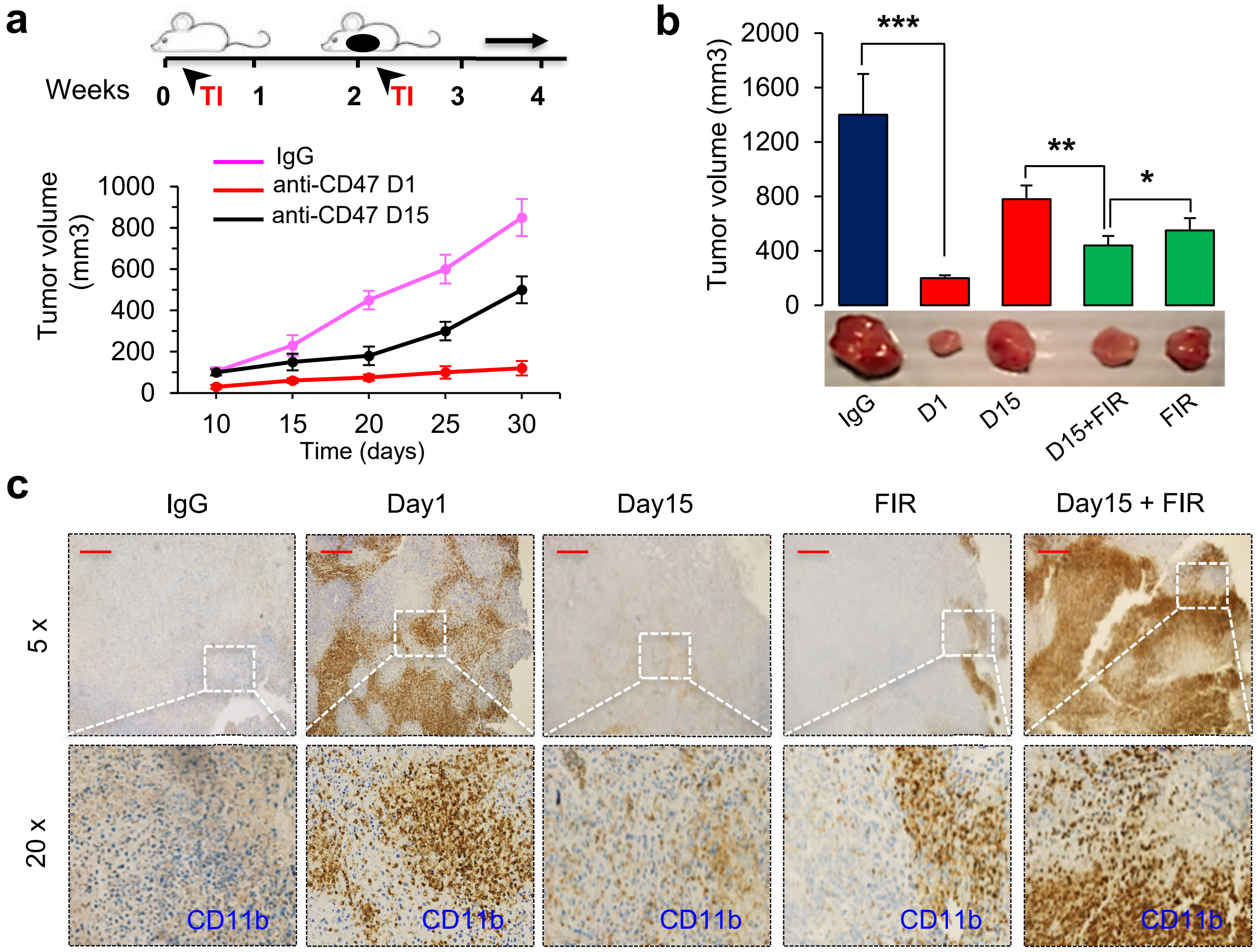

Supplementary Figure 9. Radiation-mediated control of mouse orthotopic breast tumors were synergized with anti-CD47 antibody.

(a) Syngeneic mouse breast tumors were generated by inoculation of 5 × 10^4 mouse breast tumor 4T1 cells and TI was started by injection of 50 µl PBS containing 100 µg of anti-mouse CD47 F (ab') fragments into the mammary fat pads (Day 1), into tumor tissues (Day 15) every other day with IgG injection as control with or without FIR (start at Day 15, 5 Gy/day for 4 days). Tumor sizes were measured at Day 30. (b) Representative tumor images. n = 10, *$P <$ 0. 05, **$P <$ 0. 01, ***$P <$ 0. 001). (c) IHC of $CD11b^+$ macrophages in 4T1 tumors treated with CD47 antibody, RT or combination (n = 5; representative images were shown with 5 x magnification above, or 20 × magnification bottom; scale bar = 100 µM).

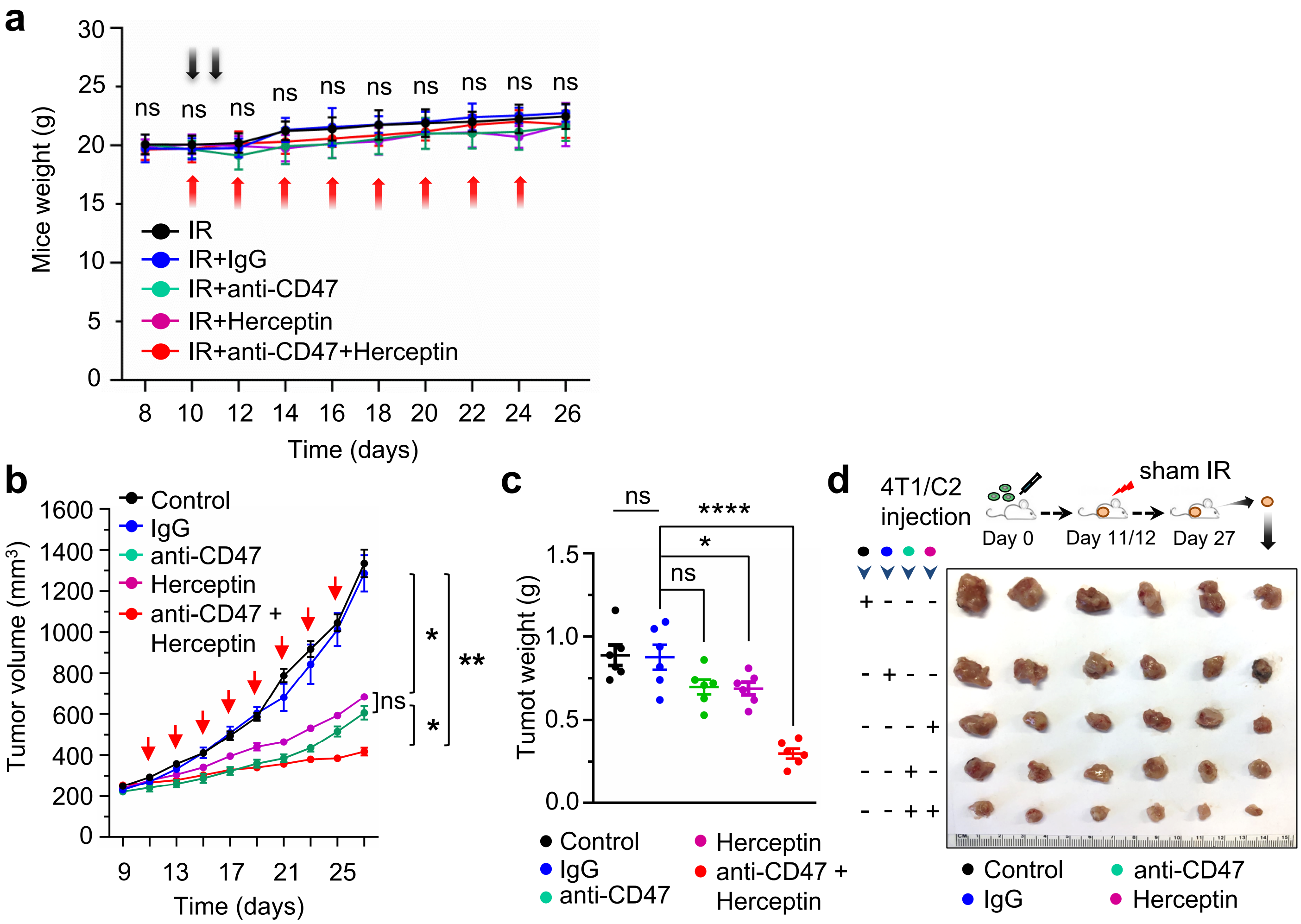

Supplementary Figure 10. Mice weight and tumor growth with indicated treatments. (a) No obvious toxicity detected by measuring mouse body weight during the course of treatment with radiation combined with anti-CD47 and anti-HER2 antibodies. BALB/c mice (n = 6 per group) transplanted mammary fat pad with 5 x 10^5 radioresistant 4T1/C2 cells. Mice were intratumorally injected with PBS, IgG, Herceptin (5 mg/kg), anti-CD47 antibody (5 mg/kg) or both antibodies every other day since day 10. All the tumors were treated with local RT with 5 Gy per fraction for 2 fractions at day 10 and day 11. Mice body weights were measured at each indicated time point, two-tailed unpaired t-test. Tumor volumes (b) and tumor weights at the end of experiments (c), and representative images (d) of mouse syngeneic breast tumors generated with radioresistant 4T1/C2 cells and treated with intratumorally injection of PBS (Control), IgG (Control), anti-CD47 antibody (5 mg/kg), Herceptin (5 mg/kg), or combination both antibodies every other day starting at day 11 (red arrow) after cell inoculation in the 4th mammary fat pad. Sham IR, sham irradiation (n = 6, Mean ± SEM, ns = not significant, *P < 0.05, **P < 0.01, ****P < 0.0001).

Abbreviation	Tumor Types	Normal (n)	Tumor (n)
BLCA	Bladder urothelial carcinoma	28	404
BRCA	Breast invasive carcinoma	291	1085
CESC	Cervical squamous cell carcinoma and endocervical adenocarcinoma	13	306
CHOL	Cholangio carcinoma	9	36
COAD	Colon adenocarcinoma	349	275
DLBC	Lymphoid Neoplasm Diffuse Large B-cell Lymphoma	337	47
GBM	Glioblastoma multiforme	207	163
LGG	Brain lower grade glioma	207	518
LIHC	Live hepatocellular carcinoma	160	369
LUAD	Lung adenocarcinoma	347	483
OV	Ovarian serous cystadenocarcinoma	88	426
PAAD	Pancreatic adenocarcinoma	171	179
READ	Rectum adenocarcinoma	318	92
STAD	Stomach adenocarcinoma	211	408
THYM	Thymoma	339	118
UCEC	Uterine Corpus Endometrial Carcinoma	91	174
UCS	Uterine Carcinosarcoma	78	57

Supplementary Table 1. Numbers and cancer types for distribution of CD47 and HER2 expression analyzed from 17 types of human cancers and their adjacent normal tissues (acquired from GEPIA database).
