# Supplementary material for: Dual blockade of CD47 and HER2 eliminates radioresistant breast cancer cells
Source: Nat Commun. 2020 Sep 14;11:4591. doi: 10.1038/s41467-020-18245-7 (PMC7490264; doi:10.1038/s41467-020-18245-7)
Supplement: Supplementary file 3 — Reporting Summary [file 41467_2020_18245_MOESM3_ESM.pdf]

nature research

Corresponding author(s): Jian Jian Li

Last updated by author(s): Jul 10, 2020

Reporting Summary

Nature Research wishes to improve the reproducibility of the work that we publish. This form provides structure for consistency and transparency in reporting. For further information on Nature Research policies, see our Editorial Policies and the Editorial Policy Checklist.

Statistics

For all statistical analyses, confirm that the following items are present in the figure legend, table legend, main text, or Methods section.

n/a	Confirmed	
☐	☒	The exact sample size (*n*) for each experimental group/condition, given as a discrete number and unit of measurement
☐	☒	A statement on whether measurements were taken from distinct samples or whether the same sample was measured repeatedly
☐	☒	The statistical test(s) used AND whether they are one- or two-sided<br>*Only common tests should be described solely by name; describe more complex techniques in the Methods section.*
☒	☐	A description of all covariates tested
☐	☒	A description of any assumptions or corrections, such as tests of normality and adjustment for multiple comparisons
☐	☒	A full description of the statistical parameters including central tendency (e.g. means) or other basic estimates (e.g. regression coefficient) AND variation (e.g. standard deviation) or associated estimates of uncertainty (e.g. confidence intervals)
☐	☒	For null hypothesis testing, the test statistic (e.g. *F*, *t*, *r*) with confidence intervals, effect sizes, degrees of freedom and *P* value noted<br>*Give P values as exact values whenever suitable.*
☒	☐	For Bayesian analysis, information on the choice of priors and Markov chain Monte Carlo settings
☒	☐	For hierarchical and complex designs, identification of the appropriate level for tests and full reporting of outcomes
☒	☐	Estimates of effect sizes (e.g. Cohen's *d*, Pearson's *r*), indicating how they were calculated

Our web collection on statistics for biologists contains articles on many of the points above.

Software and code

Policy information about availability of computer code

Data collection	1. The sgRNAs were designed using the CRISPR design software (http://crispr.mit.edu) following the instructions published by Dr. Zhang's Lab;<br>2. TRANSFAC software (Cold Spring Harbor Transcriptional Regulatory Element Database) was used to predict potential NF-κB binding motifs on the CD47 promoter region;<br>3. GEPIA database (http://gepia.cancer-pku.cn/) was used for comparing the differential expression of CD47 and HER2 among diverse types of human tumors and their corresponding non-tumor tissues.
Data analysis	1. FlowJo v10.0.7 (BD) was used for flow cytometry data analysis;<br>2. ImageJ was used for western blot band intensity analysis;<br>3. Graphpad, Microsoft Excel and SPSS were used for drawing pictures, analyzing data, and statistics, respectively;<br>4. Microsoft Power Point was used for organizing the figures;<br>5. TCGA database and Survexpress (http://bioinformatica.mty.itesm.mx:8080/Biomatec/SurvivaX.jsp) were used for Kaplan-meier survival analysis.

For manuscripts utilizing custom algorithms or software that are central to the research but not yet described in published literature, software must be made available to editors and reviewers. We strongly encourage code deposition in a community repository (e.g. GitHub). See the Nature Research guidelines for submitting code & software for further information.

Data

Policy information about availability of data

All manuscripts must include a data availability statement. This statement should provide the following information, where applicable:
- Accession codes, unique identifiers, or web links for publicly available datasets
- A list of figures that have associated raw data
- A description of any restrictions on data availability

Figures 1-6 are with raw data. Supplementary data are presented as Figures 1-10. There are no restrictions on data availability.

Field-specific reporting

Please select the one below that is the best fit for your research. If you are not sure, read the appropriate sections before making your selection.

☒ Life sciences ☐ Behavioural & social sciences ☐ Ecological, evolutionary & environmental sciences

For a reference copy of the document with all sections, see nature.com/documents/nr-reporting-summary-flat.pdf

Life sciences study design

All studies must disclose on these points even when the disclosure is negative.

Sample size	The sample sizes were determined based on our previous studies.
Data exclusions	Mice with tumors larger than 1400 mm3 or the ones that seemed uncomfortable even with a tumor smaller than 1400 mm3 were euthanized to comply with UC Davis IACUC regulations for use of vertebrate animals in research. Those were not included in our data analyses. We have no other data exclusions in this study.
Replication	1. For western blots, each experiment was repeated at least twice independently and band intensity analysis were repeated 4 times with ImageJ;<br>2. For flow cytometry analysis, each experiment was performed 3 times independently;<br>3. Immunocytochemistry, luciferase, phagocytosis, clonogenic survival, gap filling rate, tumor sphere formation and trans-well invasion assays were all repeated for at least 3 times independently.
Randomization	Mouse that were injected with tumor cells were randomly divided into groups before subsequent treatments.
Blinding	Cellular, biochemical or mice experiments were not performed in a blinded manner. Immunohistochemistry analyses were done in a blinded fashion by sending the stained pathological sections to two pathologists at UC Davis Medical Center or Ohio State University without associated patient information.

Reporting for specific materials, systems and methods

We require information from authors about some types of materials, experimental systems and methods used in many studies. Here, indicate whether each material, system or method listed is relevant to your study. If you are not sure if a list item applies to your research, read the appropriate section before selecting a response.

Materials & experimental systems

n/a	Involved in the study	
☐	☒	Antibodies
☐	☒	Eukaryotic cell lines
☒	☐	Palaeontology and archaeology
☐	☒	Animals and other organisms
☒	☐	Human research participants
☐	☒	Clinical data
☒	☐	Dual use research of concern

Methods

n/a	Involved in the study	
☒	☐	ChIP-seq
☐	☒	Flow cytometry
☒	☐	MRI-based neuroimaging

Antibodies

Antibodies used

A. Antibodies for Western blots:
1. CD47 (B6H12) was bought from Santa Cruz, Catalog # sc-12730, mouse monoclonal antibody raised from intact CD47 purified fro m placenta. More antibody information and application profile can be found in https://datasheets.scbt.com/sc-12730.pdf. References, PMID: 23292955.
2. HER2/ErbB2 (29D8) was bought from Cell Signaling, Catalog # 2165, rabbit monoclonal antibody produced by immunizing animals

with a synthetic peptide corresponding to residues surrounding tyrosine 1248 of human ErbB2 protein. More information and application profile can be found in https://media.cellsignal.com/pdf/2165.pdf. References, PMID: 27595329; PMID: 27356767; PMID: 23995768; PMID: 26150526.
3. α-tubulin, monoclonal anti-α-tubulin antibody produced in mouse, Catalog # T6074. More information can be found in https://www.sigmaaldrich.com/content/dam/sigma-aldrich/docs/Sigma/Datasheet/t6074dat.pdf and https://www.sigmaaldrich.com/catalog/product/sigma/t6074?lang=en&region=US. References, PMID: 21415014; PMID: 10339593; PMID: 21325135.
4. β-actin, monoclonal anti-β-actin antibody produced in mouse, Catalog # A5441. More information can be found in https://www.sigmaaldrich.com/content/dam/sigma-aldrich/docs/Sigma/Datasheet/6/a5441dat.pdf and https://www.sigmaaldrich.com/catalog/product/sigma/a5441?lang=en&region=US. References, PMID: 15809369 and 16904174.

B. Antibodies for Immunohistochemistry:
1. CD47 (B6H12), the same one as used for western blot listed above;
2. CD11b was bought from GeneTex, Catalog # GTX62284,rabbit monoclonal antibody, A synthetic peptide corresponding to residues in human CD11b was used as an immunogen. This antibody is no longer offered.
3. HER2/ErbB2 (29D8) was used for both western blot and IHC stain.

C. Antibodies for Immunocytochemistry:
1. CD47 (B6H12), bought from Santa Cruz, Catalog # sc-12730, mouse monoclonal antibody, was also used for ICC, information was shown above.

D. Antibodies for Flow cytometry:
1. CD47-FITC (B6H12), mouse monoclonal BALB/c IgG1, κ, was bought from BD Pharmingen, Catalog # 556045. Immunogen: purified human placental RGD-binding proteins. More information can be found in http://www.bdbiosciences.com/us/applications/research/stem-cell-research/cancer-research/human/fitc-mouse-anti-human-cd47-b6h12/p/556045. References, PMID: 28607485; PMID: 26355501; PMID: 27819324.
2. Her-2/neu APC, mouse monoclonal BALB/c IgG1, was bought from BD Pharmingen, Catalog # 340554. Immunogen: SKBR3 human breast carcinoma cells. More information can be found in http://www.bdbiosciences.com/us/applications/research/stem-cell-research/cancer-research/human/apc-mouse-anti-human-her-2neu-neu-247/p/340554. References, PMID: 23300147; PMID: 7585609; PMID: 1717984; PMID: 1346155.

E. Antibodies for Phagocytosis assay:
1. anti-human CD47 (B6H12). Fab Fragmentation Kit (G-Biosciences, Catalog # 786-272) was used in producing this antibody. The antibody purity was determined and verified by immunoblotting analysis before performing any subsequent experiments.

F. Antibodies for In vivo treatments:
1. anti-mouse CD47, MIAP301 Hybridoma, was kindly provided by Dr. William Frazier at Washington University at St. Louis. Fab Fragmentation Kit (G-Biosciences, Catalog # 786-272) was used in producing this antibody. The antibody purity was determined and verified by immunoblotting analysis before performing any subsequent experiments.
2. Herceptin (Genentech, NDC# 50242-0134-68, List # 15534) was obtained from UC Davis Comprehensive Cancer Center Pharmacy as left-over clinical medicine. More information for this therapeutic antibody can be found in https://www.gene.com/download/pdf/herceptin_dhcp.pdf.

Validation

All antibodies, except MIAP301 Hybridoma which was kindly provided by Dr. William Frazier and used for generation of anti-mouse CD47 antibody, and Herceptin (Genentech, NDC# 50242-0134-68, List # 15534) which was obtained from UC Davis Comprehensive Cancer Center Pharmacy as left-over clinical medicine, are from commercial sources and have been validated according to previous publications (see PMIDs above) and respective manufacturer's information.

Eukaryotic cell lines

Policy information about cell lines

Cell line source(s)

A. Human cell lines:
1. Breast cancer MCF-7 (ATCC® HTB-22™), MDA-MB-231 (ATCC® HTB-26™), BT474 (ATCC® HTB-20™), BT549 (ATCC® HTB-122™), SKBR3 (ATCC® HTB-30™) cell lines were all purchased from ATCC.
2. Liver cancer HepG2 (ATCC® HB-8065™) cell line was purchased from ATCC.
3. Glioblastoma U251 cell line was purchased from ATCC.
4. Colon cancer HCT116 (ATCC® CCL-247™) cell line was purchased from ATCC.

B. Mouse breast cancer 4T1 (ATCC® CRL-2539™) cell line was purchased from ATCC.

C. Each radioresistant cancer cell line was derived from its corresponding counterpart parental cell line by application of clinical mimic radiation with fractionated doses (2 Gy x 20 - 30 fractions). After fractionated radiation, multiple survived clones were selected out and verified by comparison of their radioresistance to parental cell lines.

D. The radioresistant HER2+ and HER2- BCSCs were sorted out via flow cytometry from the radioresistant MCF7/C6 population.

Authentication

All human and mouse breast cancer cell lines used in this study were authenticated by the supplier, and human breast cancer cells are regularly authenticated by STR.

Mycoplasma contamination

All cell lines were tested negative for mycoplasma contamination with MycoSensor PCR Assay kit (Agilent technologies, Catalog # 302108; more information can be found in https://www.genomics.agilent.com/article.jsp?

	crumbAction=push&pageId=536) in the mid- and late-stages of study.
Commonly misidentified lines (See ICLAC register)	No commonly misidentified cell lines were used in this study.

Animals and other organisms

Policy information about studies involving animals; ARRIVE guidelines recommended for reporting animal research

Laboratory animals	1, NSG (NOD SCID gamma; NOD.Cg-Prkdcscid Il2rgtm1Wjl/SzJ) mice (Jackson Lab # 005557), eight-week-old, female.<br>2, BALB/c mice (Jackson Lab #000651), eight weeks old, female.
Wild animals	This study did not use wild animals.
Field-collected samples	This study did not use field-collected samples.
Ethics oversight	Animal care, study approval, and protocol execution were all conducted under the oversight of the UC Davis IACUC.

Note that full information on the approval of the study protocol must also be provided in the manuscript.

Clinical data

Policy information about clinical studies

All manuscripts should comply with the ICMJEguidelines for publication of clinical research and a completedCONSORT checklist must be included with all submissions.

Clinical trial registration	N/A
Study protocol	N/A
Data collection	N/A
Outcomes	N/A

Flow Cytometry

Plots

Confirm that:

- [x] The axis labels state the marker and fluorochrome used (e.g. CD4-FITC).
- [x] The axis scales are clearly visible. Include numbers along axes only for bottom left plot of group (a 'group' is an analysis of identical markers).
- [x] All plots are contour plots with outliers or pseudocolor plots.
- [x] A numerical value for number of cells or percentage (with statistics) is provided.

Methodology

Sample preparation	Described in the Methods section.
Instrument	Becton Dickinson Canto
Software	FlowJo v10.0.7 (BD)
Cell population abundance	Cell sorting was not applied in this project.
Gating strategy	Forward vs side scatter (FSC-A versus SSC-A) was used as gating strategy to identify the population of interest and exclude the dead cells and cells debris. The FSC-A/FSC-H were used to remove the doublets from total cell population. Positive cells were gated out based on the gating of unstained cells and single stained cells.

- [x] Tick this box to confirm that a figure exemplifying the gating strategy is provided in the Supplementary Information.
